# Nutritional Iron Deficiency Anemia: Magnitude and Its Predictors among School Age Children, Southwest Ethiopia: A Community Based Cross-Sectional Study

**DOI:** 10.1371/journal.pone.0114059

**Published:** 2014-12-01

**Authors:** Amare Desalegn, Andualem Mossie, Lealem Gedefaw

**Affiliations:** 1 Department of Biomedical Sciences, College of Public Health and Medical Sciences, Jimma University, Jimma, Ethiopia; 2 Department of Medical Laboratory Science and Pathology, College of Public Health and Medical Sciences, Jimma University, Jimma, Ethiopia; CUNY, United States of America

## Abstract

**Background:**

Iron deficiency anemia (IDA) is a global public health problem among school age children, which retards psychomotor development and impairs cognitive performance. There is limited data on prevalence and risk factors for IDA.

**Objective:**

The aim of this study was to determine the prevalence, severity, and predictors of nutritional IDA in school age children in Southwest Ethiopia.

**Methodology:**

A community based cross-sectional study was conducted in Jimma Town, Southwest Ethiopia from April to July 2013. A total of 616 school children aged 6 to 12 years were included in the study using multistage sampling technique. A structured questionnaire was used to collect sociodemographic data. Five milliliter venous blood was collected from each child for hematological examinations. Anemia was defined as a hemoglobin level lower than 11.5 g/dl and 12 g/dl for age group of 5–11 years and 12–15 years, respectively. Iron deficiency anemia was defined when serum iron and ferritin levels are below 10 µmol/l and 15 µg/dl, respectively. Moreover, fresh stool specimen was collected for diagnosis of intestinal parasitic infection. Stained thick and thin blood films were examined for detection of *Plasmodium* infection and study of red blood cell morphology. Dietary patterns of the study subjects were assessed using food frequency questionnaire and anthropometric measurements were done. Data were analyzed using SPSS V-20.0 for windows.

**Result:**

Overall, prevalence of anemia was 43.7%, and that of IDA was 37.4%. Not-consuming protein source foods [AOR = 2.30, 95%CI(1.04,5.14)], not-consuming dairy products [AOR = 1.83, 95%CI(1.14,5.14)], not-consuming discretionary calories [AOR = 2.77, 95%CI(1.42,5.40)], low family income [AOR = 6.14, 95%CI(2.90,12.9)] and intestinal parasitic infections [AOR = 1.45, 95%CI(1.23, 5. 27)] were predictors of IDA.

**Conclusion:**

Iron deficiency anemia is a moderate public health problem in the study site. Dietary deficiencies and intestinal parasitic infections were predictors of IDA. Therefore, emphasis should be given to the strategies for the prevention of risk factors for IDA.

## Introduction

Anemia is a condition, characterized by reduction in the red blood cell volume or decrease in the concentration of hemoglobin (Hb) in the blood [1]. Prevalence of anemia is high in the developing world. Its causes are multifactorial, ranging from micronutrient deficiencies such as iron, folate and vitamin B_12_ to infectious diseases such as malaria and worm infections [2], [3]. According to the joint report of World Health Organization (WHO) and Center for Disease Control and prevention (CDC) in 2008, the global prevalence of anemia in school age children was 25.4% [4]. The normal proliferation and differentiation of hematopoietic stem cells in the bone marrow requires several essential nutrients, such as iron, folate, and vitamin B_12_ [5]. Iron is a micronutrient that is essential for cell growth and differentiation [6]. In the human body, when iron intake and absorption no longer meets the need of normal iron turnover and losses, and iron stores are exhausted then insufficient amounts of iron will be delivered to transferrin, which is the circulating transport protein of iron. This results in decreased transferrin saturation and when the depletion is sufficient to affect Hb synthesis, a state of iron deficiency anemia occurs [7]. Iron deficiency anemia is the most severe stage of iron deficiency in which Hb concentration falls below a statistically defined threshold [8].

Globally, 50% of anemia is attributable to iron deficiency and accounts for approximately 841,000 deaths annually worldwide. Africa and parts of Asia bear 71% of the global mortality burden; North America represents 1.4% of the total morbidity and mortality associated with iron deficiency anemia [9]. According to WHO's report, in 2001, prevalence of IDA among school age children in industrialized countries was 5.9%. However, in developing countries, the prevalence was 48.1%. Iron deficiency in children is known to retard psychomotor development and impair cognitive performance, increased morbidity from infectious disease, and decrease work capacity. Moreover, iron deficient individuals have increased absorption capacity of divalent heavy metals, including toxic metals such as lead and cadmium, apparently increasing risk of metal poisoning [10].

Iron deficiency anemia is the most common nutritional deficiency in many developing countries with major health, social and economic consequences. Ethiopia, as one of the developing countries, shares the burden. Despite this, data on prevalence and risk factors of nutritional iron deficiency anemia particularly in school age children in Ethiopia is limited. Therefore, this study was aimed at determining prevalence, severity and predictors of nutritional IDA among school-age children in Southwest Ethiopia.

## Materials and Methods

### Study design, population and sampling techniques

A community based cross-sectional study was conducted in Jimma Town, Southwest Ethiopia from April to July 2013. A total of 616 school children aged from 6 to 12 years available at the time of study were included in the study. Multistage sampling was used to select the study participants. First, a list of clusters was established by using Kebeles (the smallest administrative unit in Ethiopia). From the total 13 Kebeles (clusters) of the town, 6 Kebeles were selected randomly by lottery method. The sampling frame was prepared for the household in the selected Kebeles and sample was proportionally allocated to the selected Kebeles. By using systematic random sampling method households were identified in each of the selected Kebele. In each of the selected Kebele, the first household was selected randomly and the next household was determined by sampling interval. Sampling interval (K) was calculated by dividing the total household of each Kebele to the sample size. One child was interviewed from each of the selected households. If more than one child dwells in a household, one was chosen by the lottery method. School age children with history of severe illness, and recent medication use including treatment for anemia for the last two weeks prior to data collection were excluded from the study.

### Data collection

Socio-demographic, economic and data on dietary patterns was collected using pretested questionnaire. Moreover, anthropometric measurements and laboratory data were collected. The data collection staff includes clinical nurses and laboratory technologists, under supervision of general practitioners. One day training was given to the data collectors and supervisors prior to data collection.

### Dietary assessment

Dietary patterns of the children were assessed using the food frequency questionnaires (FFQ). Food frequency questionnaires were modified from the Helen Keller International FFQ and used previously in Jimma, Ethiopia to estimate dietary practices of adolescents [11]. The list of food items in the questionnaire were grouped into seven food categories (grains, vegetables, fruits, protein source food, fat/oils and discretionary calories) based on the availability of the food items commonly consumed in the study area and according to the MyPyramid classification for healthy eating[12]. The food frequency questionnaire (FFQ) was designed to obtain qualitative information about the usual food consumption patterns of children on certain food items or groups of food items consumed during a specific time [13]–[14]. Children were coded as a “consumer” of a food item if they have consumed the food item of the above group at least once per week [15]. Two measures called as dietary diversity (DD) [16]–[17] and consumption of animal source foods (AFS) [18]–[20] were used to determine the dietary practices of children. Then a dietary diversity score (DDS) was constructed by counting the intake of the food groups over a period of one week based on the definition that it is the sum of food groups consumed over the reference period [11], [21]. For example, children who consumed one item from each of the food groups at least once during the week would have the maximum DDS of seven. The DDS was converted into tertiles and the highest tertile was used to define “high” dietary diversity score, while the two lower tertiles combined were labeled as “low” dietary diversity score and the same rule and method was used for animal source food (AFS) [11].

### Anthropometric measurements

Anthropometric parameters measured include: height and weight were done. Body weight was measured to the nearest 0.1 kg on a battery powered digital scale and height was measured to the nearest 0.1 cm using a fixed base portable wooden length measuring board with a sliding head bar [22]. The following indicators: height-for-age z scores (HAZ), weight-for-age z scores (WAZ), and body mass index-for-age z score (BMIZ) were used to assess the nutritional status of school age children [23]. Children who fall below minus two standard deviations (-2SD) and -3SD from the median of the reference population were considered as moderately and severely malnourished, respectively [24].

### Blood sample collection and analysis

Each participated child provided approximately 5 ml of venous blood sample. Two (2 ml) was drawn into the EDTA containing test tube and used for the complete blood count and peripheral blood smear preparation. Hematological parameters: hemoglobin (Hb), hematocrit, mean cell volume (MCV), mean cell hemoglobin (MCH), mean cell hemoglobin concentration (MCHC), red blood cell count (RBC), and white blood cell count (WBC) were performed using ABX PENTRA ML hematology analyzer (HORIBA ABX SAS, France). Three ml of venous blood was drawn into plane test tube and serum was separated for biochemical analysis. Serum sample was kept on −20°C prior to analysis as per the manufacturer's instruction. Serum iron and ferritin were measured using ABX HORIBA PENTRA 400 clinical chemistry analyzer (HORIBA ABX SAS, France). The specimens were analyzed in hematology laboratory of Jimma University.

#### Definition of anemia

School-age children with Hb levels lower than 11.5 g/dl and 12 g/dl was considered as anemic for age ranges from 5–11 and 12–15 years old, respectively. Mild anemia was defined as the Hb concentration between 10–11.9 g/dl for 12–15years and Hb concentration of 10–11.4 g/dl for 5–11years school children. Moderate anemia was defined as the Hb concentration between 7–9.9 g/dl and severe anemia was defined as Hb concentration lower than 7 g/dl. Iron deficiency anemia was defined when serum iron and ferritin levels are below 10 µmol/l and 15 µg/dl, respectively [10], [25]. WBC count greater than 10,000 cells/mm^3^ was used as an indicator for the presence of a possible infection or inflammation [26].

### Blood film and stool examination

Children were screened for hemoparasites and worm infections to assess their impact on IDA. Malaria was diagnosed by microscopic examination of Giemsa (10%) stained thick and thin blood films. For intestinal parasitic infection approximately 4 g of fresh stool sample was collected from each child using clean, leak proof stool cup, and then transferred to Medical Parasitology laboratory of Jimma University. The samples were processed using direct and formol ether concentration techniques, following standard procedures. Red blood cell morphology was done on wright stained thin blood smears. Morphological classification of anemia was done using microscopic examination of red blood cell morphology and values of red cell indices.

### Data quality assurance

To assure the quality of the data generated, standard operating procedures were followed during specimen collection and all laboratory procedures. The laboratory analysis were performed according to the manufacturers' instructions. Control reagents were used for the hematology and clinical chemistry analyzers to check the reproducibility of the results. Training was given for the data collectors to minimize technical and observation bias.

### Data analysis

Data were entered into computer using EPI data version 3.1, cleaned and exported to SPSS Version 20 for analysis. Descriptive statistics were employed to summarize the data. Multivariable logistic regression analysis was done to identify the independent predictors of nutritional iron deficiency anemia. All variables with a p value less than 0.05 were considered as statistical significance. Anthropometric data were entered and processed by WHO Anthro Plus version 1.0.4 software.

### Ethics statement

Ethical clearance was obtained from Jimma University Ethical Review Committee. Permission was sought from the Jimma Town municipality. Written informed consent was obtained from the guardians of the children and additional oral assent was obtained from 7–12 years old children after describing the benefits and risks of the study. Children with intestinal parasitic infections and those with hematological values below the reference ranges were referred to health professionals in Jimma University Specialized Hospital for possible interventions.

## Results

### Socio demographic characteristics

A total of 586 children, (53.8% female and 46.2% male) had participated in the study, with a response rate of 95%. The mean (±SD) age of the children was 8.9 years (±2.01). With regard to educational status of children's parents, 260 (44.4%) of their mothers and 213 (36.3%) of their father had primary education. The majority of the children's parents, 234 (39.9%) had monthly income greater than 1,000 ETB (1$US = 19.94 Ethiopian Birr) and 152 (25.9%) had monthly income less than 500 ETB (Table1).

**Table 1:** Bivariable analysis of socio-demographic characteristics and iron deficiency anemia among school age children, Jimma Town, Southwest Ethiopia, 2013 (n = 586).

Variable		Total	Iron deficiency anemia		P-value	COR (95%CI)
		(n = 586) n (%)	Yes (n = 220) n (%)	No (n = 366) n (%)		
Age	6–9	**317(54.1)**	132(41.6)	185(58.4)	**0.026**	1.47(1.05, 2.06)
	10–12	269 (45.9)	88(32.7)	181(67.3)		1
Sex	Male	271 (46.2)	108(39.9)	163(60.1)	0.284	1.2(0.86, 1.68)
	Female	**315 (53.8)**	112(35.6)	203(64.4)		1
Family size	Low(1–4)	**240(41.0)**	89(37.1)	151(62.9)	0.56	1.18(0.68, 2.06)
	Second(5)	160(27.3)	61(38.1)	99(61.9)	0.48	1.23(0.69, 2.21)
	Middle(6)	114(19.5)	46(40.4)	68(59.6)	0.34	1.35(0.73, 2.51)
	Highest (7)	72(12.3)	24(33.3)	48(66.7)		1
Income in tertile*	<500 ETB	152(25.9)	99(65.1)	53(34.9)	**<0.0001**	11.8(6.55, 21.24)
	500–1000 ETB	200(34.1)	91(45.5)	109(54.5)	**<0.0001**	5.3(3.02,9.22)
	>1001 ETB	**234(39.9)**	30(12.8)	204(87.2)		1
Father's educational status	Illiterate	28(4.8)	10(35.7)	18(64.3)	0.235	1.7(0.713, 3.97)
	Primary	**213(36.3**)	84(39.4)	129(60.6)	**<0.0001**	1.97(1.24, 3.13)
	Secondary	196(33.4)	89(45.4)	107(54.6)	**<0.0001**	2.5(1.58, 4.01)
	Tertiary	149 (25.4)	37(24.8)	112(75.2)		1
Father's occupation	Merchant	104(17.7)	35(33.7)	69(66.3)	0.26	0.5(0.15, 1.69)
	Daily laborer	116(19.8)	54(46.6)	62(53.3)	0.16	0.5(0.19,1.33)
	Civil servants	**206(35.2)**	69(33.5)	137(66.5)	0.77	0.9(0.34,2.25)
	No work	20(3.4)	6(30)	14(70.0)	0.14	0.5(0.2,1.27)
	Owen private	48(8.2)	16(33.3)	32(66.7)	0.99	0.0(0.00, 0.00)
	Others**	92(15.7)	40(43.5)	52(56.5)		1
Mother's educational status	Illiterate	83(14.2)	30(36.1)	53(63.9)	0.90	2.01(0.89, 4.49)
	Primary	**260(44.4)**	106(40.8)	154(59.2)	0.01	2.44(1.19, 4.98)
	Secondary	193(32.9)	73(37.8)	120(62.2)	0.04	2.16(1.04, 4.47)
	Tertiary	50(8.5)	11(22.0)	39(78.0)		1
Mother's occupation	Merchant	**131(22.4)**	49(37.4)	82(62.6)	0.82	0.86(0.23, 3.16)
	Daily laborer	120(20.5)	50(41.7)	70(58.3)	0.52	0.73(0.28,1.89)
	Civil servants	121(20.6)	40(33.1)	81(66.9)	0.78	0.87(0.34,2.26)
	House wife/None	108(18.4)	44(40.7)	64(59.3)	0.30	0.60 (0.23,1.58)
	Owen private	39(6.7)	12(30.8)	27(69.2)	1.00	1.98(0.00,)
	Others**	67(11.4)	25(37.3)	42(62.7)		1

**Key**: * 1 US $ = 19.94 Ethiopian Birr (ETB), **others  =  Farming, Factory worker, Hand craft and Non-governmental.

### Dietary habit, anthropometric measurements, and intestinal parasitic infection

All of the children consume grain food sources at least once in a week. However; vegetables, fruits, dairy products, protein, oils, or fat and discretionary calorie food sources were not consumed by the children as that of the grain food sources. Majority (71%) of the children had a low dietary diversity score and the remaining (29%) had a high dietary diversity score. The mean weight and height of the children were 27.3 (±5.84) kg and 131 (±0.12) cm, respectively. Thinness and severe thinness were recorded in 57 (9.7%) and 50 (8.5%) of the children, respectively. Overall, 134 (33.9%) of the children had intestinal parasitic infections. *Hookworm* was the most 76 (56.8%) prevalent intestinal parasite detected followed by *Trichuris trichiuria* 33 (24.6%) and *Ascaris lumbricoides* 25 (18.6%). No malaria infections were detected from the blood films (Table 2).

**Table 2:** Bivariable analysis of dietary habit, anthropometric measurements, and intestinal parasite infection with iron deficiency anemia among school age children, Jimma Town, Southwest Ethiopia, 2013 (n = 586).

Variable		Total	Iron deficiency anemia		P-value	COR (95%CI)
		(n = 586) n (%)	Yes (n = 220) n (%)	No (n = 366) n (%)		
Vegetables	Non Consumer	86(14.7)	50(58.1)	36(41.9)	**<0.0001**	2.69(1.69, 4.29)
	Consumer	**500(85.3)**	170(34.0)	330(66.0)		1
Fruits	Non -Consumer	197(33.6)	100(50.8)	97(49.2)	**<0.0001**	2.31(1.62, 3.28)
	Consumer	**389(66.4)**	120(30.8)	269(69.2)		1
Dairy products	Non -Consumer	216(36.9)	130(60.2)	86(39.8)	**<0.0001**	3.72(2.72, 5.31)
	Consumer	**370(63.1)**	90(24.3)	280(75.7)		1
Protein source foods	Non -Consumer	44(7.5)	29(65.9)	15(34.1)	**<0.0001**	3.55(1.85, 6.79)
	Consumer	**542(92.5)**	191(35.2)	351(64.8)		1
Oils	Non -Consumer	**417(71.2)**	188(45.1)	229(54.9)	**<0.0001**	3.52(2.28, 5.41)
	Consumer	169(28.8)	32(18.9)	137(81.1)		1
Discretionary calories	Non -Consumer	**350(63.8)**	179(51.1)	171(48.9)	**<0.0001**	3.83(2.57, 5.69)
	Consumer	236(36.2)	41(17.4)	195(82.6)		1
DDS*	Low consumer	**416(71.0)**	193(46.4)	223(53.6)	**<0.0001**	4.56(2.91, 7.29)
	High consumer	170(29.0)	27(15.9)	143(84.1)		1
AFS**	Low consumer	**410 (70.0)**	190(46.3)	220(53.7	**<0.0001**	4.21(2.71, 6.51)
	High consumer	176 (30.0)	30(17.0)	146(83.0)		1
HFA(z-score) ***	Stunted	95(16.2)	34(35.8)	61(64.2)	0.617	1.23(0.54, 2.80)
	Severely stunted	27(4.6)	9(33.3)	18(66.7)	0.814	1.11(0.45, 2.75)
	Normal	**464(79.2)**	177(38.1)	287(61.9)		1
WFA(z-score)****	Underweight	32(5.5)	21 (65.6)	11(34.4)	0.238	0.63 (0.29, 1.36)
	Severely underweight	28(4.8)	13 (46.4)	15(53.6)	0.137	2.20 (0.78, 6.24)
	Normal	**526(89.8)**	186 (35.4)	340(64.6)		1
BMIA(z-score) *****	Over weight	82(14.0)	33(40.2)	49(59.8)	**0.004**	0.42(0.23, 0.76)
	Obesity	2(0.3)	1(50.0)	1(50.0)	0.125	0.57(0.28, 1.16)
	Thinness	57(9.7)	29(50.9)	28(49.1)	0.911	0.85(0.05, 14.4)
	Severe thinness	50(8.5)	27(54.0)	23(46.0)	0.747	0.88(0.41, 1.89)
	Normal	**395(67.4)**	130(32.9)	265(67.1)		1
Intestinal parasite infection	Yes	134(33.9)	30(22.4)	104(77.6)	**0.025**	2.30(1.75, 3. 48)
	No	**452(77.1)**	190(42.0)	262(58.0)		1

**Key**: *DDS  =  Dietary diversity score, **AFS  =  Animal sours food score, ***HFA  =  Height for age, ****WFA  =  Wight for age, *****BMIA  =  Body mass index for age.

### Prevalence and severity of iron deficiency anemia

Children's Hb level was used to determine the prevalence of anemia. The overall prevalence of anemia was 256 (43.7%) with mean Hb value of 12 (±2.4) g/dl. Among anemic children, 15.6%, 49.2%, and 35.2% had severe, moderate and mild anemia, respectively. Nutritional iron deficiency anemia was diagnosed in 220 (37.3%) of the children. The mean values of serum iron and serum ferritin were 12.4 (±7.3) µmol/l and 10.5 (±2.2) µg/dl, respectively. Microscopic examination of the peripheral blood film showed normocytic normochromic picture in 318 (54.3%), microcytic hypochromic picture in 213 (36.3%) and normocytic hypochromic picture in 55 (9.4%) of school age children.

### Predictors of iron deficiency anemia

Both bivariable and multivariable logistic regression analysis were done to identify the independent predictors of IDA in children. All the variables were analyzed in bivariable logistic regression analysis and then those with p - value <0.25 and those believed to have biological relation with iron deficiency anemia were candidate for multivariable logistic regression analysis. Accordingly, children with low family monthly income, not consuming protein source foods, not consuming dairy products, not consuming discretionary calories and having an intestinal parasite infection were identified as predictors of IDA among the children (Table 3).

**Table 3:** Multivariable logistic regression analysis for the association of different variables with iron deficiency anemia among school age children, Jimma Town, Southwest Ethiopia, 2013 (n = 586).

Variable		Total	Iron deficiency anemia		P-value	AOR (95%CI)
		(n = 586) n (%)	Yes (n = 220) n (%)	No (n = 366) n (%)		
Age	6–9	317(54.1)	132(41.6)	185(58.4)	0.23	1.31(0.84, 2.01)
	10–12	269 (45.9)	88(32.7)	181(67.3)		1
Income in tertile*	<500 ETB	152(25.9)	99(65.1)	53(34.9)	**<0.0001**	6.14(2.90, 12.9)
	500–1000 ETB	200(34.1)	91(45.5)	109(54.5)	**<0.0001**	3.38(1.75, 6.5)
	>1001ETB	**234(39.9)**	30(12.8)	204(87.2)		1
Vegetables	Non Consumer	86(14.7)	50(58.1)	36(41.9)	0.09	1.68(0.92, 3.09)
	Consumer	**500(85.3)**	170(34.0)	330(66.0)		1
Fruits	Non -Consumer	197(33.6)	100(50.8)	97(49.2)	0.16	0.69(0.42, 1.15)
	Consumer	**389(66.4)**	120(30.8)	269(69.2)		1
Dairy products	Non -Consumer	216(36.9)	130(60.2)	86(39.8)	**0.01**	1.83(1.14, 5.14)
	Consumer	**370(63.1)**	90(24.3)	280(75.7)		1
Protein source foods	Non -Consumer	44(7.5)	29(65.9)	15(34.1)	**0.04**	2.30(1.04, 5.14)
	Consumer	**542(92.5)**	191(35.2)	351(64.8)		1
Oils	Non -Consumer	**417(71.2)**	188(45.1)	229(54.9)	0.44	1.32(0.66, 2.63)
	Consumer	169(28.8)	32(18.9)	137(81.1)		1
Discretionary calories	Non -Consumer	**350(63.8)**	179(51.1)	171(48.9)	**0.03**	2.77(1.42, 5.40)
	Consumer	236(36.2)	41(17.4)	195(82.6)		1
DDS**	Low consumer	**416(71.0)**	193(46.4)	223(53.6)	0.12	0.46(0.18, 1.20)
	High consumer	170(29.0)	27(15.9)	143(84.1)		1
AFS***	Low consumer	**410 (70.0)**	190(46.3)	220(53.7	0.17	1.48(0.85, 2.59)
	High consumer	176 (30.0)	30(17.0)	146(83.0)		1
WFA(z-score)****	Underweight	32(5.5)	21 (65.6)	11(34.4)	0.73	0.81(0.25, 2.61)
	Severely underweight	28(4.8)	13 (46.4)	15(53.6)	0.20	2.61(0.59, 11.4)
	Normal	**526(89.8)**	186 (35.4)	340(64.6)		1
BMIA(z-score) *****	Over weight	82(14.0)	33(40.2)	49(59.8)	0.81	0.89(0.38, 2.15)
	Obesity	2(0.3)	1(50.0)	1(50.0)	0.45	1.47(0.55,3.95)
	Thinness	57(9.7)	29(50.9)	28(49.1)	0.63	2.11(0.11, 41.8)
	Severe thinness	50(8.5)	27(54.0)	23(46.0)	0.47	1.48(0.52,4.25)
	Normal	**395(67.4)**	130(32.9)	265(67.1)		1
Intestinal parasite infection	Yes	134(33.9)	30(22.4)	104(77.6)	**0.001**	1.45(1.23, 5. 27)
	No	**452(77.1)**	190(42.0)	262(58.0)		1

**Key**: * 1 US$  =  19.94 Ethiopian Birr (ETB), **DDS  =  Dietary diversified score, *** AFS  =  Animal food sources score, **** WFA  =  Wight for age, ***** BMIA  =  Body mass index for age.

## Discussion

Micronutrient deficiency is a major contributor to childhood morbidity and mortality. Iron deficiency is a global nutritional problem, which mainly affects infants, children and women of childbearing age [10]. The overall prevalence of anemia in this study was 43.7%. Nutritional IDA was diagnosed in 37.3% of the children.

This shows that, anemia is a severe public health problem among school age children. The possible explanation for severe anemia among children in the present study could be due to dietary deficiency for economic constraints and high burden of intestinal parasitic infection. The prevalence of anemia in the current study is in harmony with the study conducted in most developing countries (46%) [27]; in five African countries (40%) [28]; and in Northern Ethiopia (40.5%) [29]. The prevalence of anemia in the current study is slightly higher compared to the prevalence of anemia in Asendabo Town, Southwest of Ethiopia (39.1%) [30]; Cote Divoire (39.4%) [31]; Leyte, Philippines (36.9%) [32]; and in Vietnamese (36.4%) [33]. However, it is very much less as compared to the report from the Tanga region of Tanzania (79.6%) [34]; Kilimanjaro region of Tanzania (70%) [35]; and from Abia State of Nigeria (83%) [36]. Geographical, economic, seasonal and behavioral variations of factors across these different settings may account for the difference.

The current prevalence of IDA in children was 37.5%, which indicate iron deficiency anemia is a moderate public health problem. The current prevalence of IDA is lower than a study done in Abia State of Nigeria (77.8%) [36]. This high prevalence of IDA in children of Nigeria might be due to the high intestinal parasitic infection and low socioeconomic conditions characterized by inadequate water supply and poor sanitary conditions. The current prevalence of IDA is higher as compared to studies done in: Kazakhstan (32.4%) in 2003 [37] and 13% in 2004 [26], rural area of Andhra Pradesh, India (23.1%) [38] and Morocco (20.4%) [39].

From multivariable logistic regression analysis, a significant association is obtained between iron deficiency anemia and family monthly income; low consumption of protein, discretionary and dairy source foods; and intestinal parasitic infection. Children whose parents has low average monthly income (<500 ETB) are six times more likely to be anemic than those with higher family income. Similar reports have shown that, children living with families having low income are at greater risk of IDA compared to those with higher income. This is because parents may not have enough money to spend on food. Low income often correlates with limited access to food and poor sanitation [40]–[42].

Children who do not consume protein source foods are 2.3 times more likely to be anemic than those who consume protein source foods. Moreover, those who do not consume discretionary calories and dairy food products are more likely to be anemic compared to consumers. This indicates poor nutritional intake, especially less consumption of protein, discretionary and dairy source foods were the primary factor of iron deficiency anemia in the children, probably related with low family income. Children who lack access to those varieties of food items are eventually deteriorate further in their dietary quality [43]–[48].

On the other hand, children with intestinal parasitic infections were significantly more anemic than those children who do not infected by intestinal parasites. This shows intestinal parasitic infections are strongly associated with iron deficiency anemia. This is because most of the infections were due to *Hook worm* and *Trichuris trichiuria*. *Hook worm* and *Trichuris trichiuria* infections cause blood lose in the host which may cause iron deficiency anemia.

The major strength of the study is the fact that adequate and representative samples were included. On the other hand, as it is a cross-sectional survey, it suffers from the usual “egg or chicken” dilemma. Moreover, C-reactive protein test was not used to screen school children for the presence of infection or inflammation. Also, our study did not measure serum vitamin B_12_ and folate levels, which could help identify specific causes of anemia in the children.

In conclusion, iron deficiency anemia is a moderate public health problem among the school-age children in Jimma Town. Not-consuming protein source foods, not-consuming dairy products not-consuming discretionary calories, low family income and intestinal parasitic infections were identified as independent risk factors of IDA. Therefore, emphasis should be given to reduce the risk factors of IDA. Further longitudinal studies with long term follow-up are needed.
