# Correction: Nutritional Iron Deficiency Anemia: Magnitude and Its Predictors among School Age Children, Southwest Ethiopia: A Community Based Cross-Sectional Study

**DOI:** 10.1371/journal.pone.0202380

**Published:** 2018-08-09

**Authors:** Amare Desalegn Wolide, Andualem Mossie, Lealem Gedefaw

The first author’s name is incorrect. The correct name is: Amare Desalegn Wolide. The correct citation is: Desalegn Wolide A, Mossie A, Gedefaw L (2014) Nutritional Iron Deficiency Anemia: Magnitude and Its Predictors among School Age Children, Southwest Ethiopia: A Community Based Cross-Sectional Study. PLoS ONE 9(12): e114059. https://doi.org/10.1371/journal.pone.0114059
